# Analysis of Student Perceptions of a Newly Developed Integrative System Course Model

**DOI:** 10.1007/s40670-023-01834-8

**Published:** 2023-08-01

**Authors:** Amber Sechelski, Ritvik Bhattacharjee, Austin Reynolds, Mary Manis, Hatem A. Elshabrawy, Yuan Zhao

**Affiliations:** 1 Division of Educational Affairs, College of Osteopathic Medicine, Sam Houston State University, Conroe, TX USA; 2 College of Osteopathic Medicine, Sam Houston State University, Conroe, TX USA; 3 Department of Primary Care and Clinical Medicine, College of Osteopathic Medicine, Sam Houston State University, Conroe, TX USA; 4 Trillium Educational Partners, 221 Trillium Park Loop, Conroe, TX USA; 5 Department of Molecular and Cellular Biology, College of Osteopathic Medicine, Sam Houston State University, Conroe, TX USA

**Keywords:** Curriculum integration, Course evaluation, Qualitative analysis

## Abstract

**Background:**

During Spring 2021, we piloted a course model that integrated the immune system and HEENT (head, eyes, ears, nose, and throat) by concurrently presenting them in the context of clinical cases. Immune system topics (e.g., infection, cancer) were tied to their manifestations in the HEENT system, and concepts from both systems were consolidated in weekly case-based learning and small group discussion (CBL/SGD) sessions.

**Methods:**

To evaluate students’ perceptions of the effectiveness of this model, we administered to the class a voluntary survey containing closed- and open-ended items; conducted a focus group of 10 students selected via convenience sampling; and employed a mixed approach to analyze the resulting data, including multiple qualitative methods.

**Results:**

Thirty-nine of 74 students completed the survey (53% response rate). In response to the item related to overall effectiveness of using CBL/SGD for system integration, nearly half (48.72%) of these students rated the overall effectiveness as average. Constant comparison analysis of the qualitative data revealed three major themes–student satisfaction with integration of immunology and HEENT, content and time involved in CBL/SGD, and suggestions for improvement–and classical content analysis revealed the relative importance of these themes. Participants held positive and negative perceptions, expressed concerns regarding CBL/SGD (e.g., its helpfulness, complexity), and made suggestions for improvement of integration.

**Conclusions:**

Using multiple methods allowed us to gain a deeper understanding of students’ perceptions of the new course model, and we have taken actions to improve course quality in the future.

## Introduction

Over the past two decades, both the content of medical education and the structure of medical school curricula have evolved in response to the dynamic nature of healthcare. Students are increasingly expected to achieve milestones in competencies from a broader scope of cross-disciplinary domains [1]. Medical educators are continuously exploring innovative teaching modalities to build connections between foundational and clinical sciences so students can apply integrated knowledge to understand health and disease and improve clinical performance [2, 3]. As a brand-new medical school with a mission of training osteopathic physicians who can serve the needs of rural and underserved communities, we had a unique opportunity to develop an innovative and mission-focused system course that specifically integrated the immune system with HEENT (head, eyes, ears, nose, and throat).

Among all foundational science disciplines, immunology is one of the challenging subjects to understand, and in which to find clinical relevance for first-year medical students, due to its mechanistic intricacies [4, 5]. To provide focused attention to the topic, it is usually offered as an independent foundational science course/block or combined with topics such as microbiology and hematology, based on a comprehensive review of online medical school curriculum data (unpublished data). Clinical sciences are often introduced in stand-alone system courses focusing on comprehensive learning pertaining to a single anatomical or physiological system that may not be optimal for teaching complex clinical problems involving multiple organs in multiple systems [6]. The contiguous mucosal membranes of the eyes, ears, nose, mouth, and throat constantly engage with microbes, allergens, and other antigens, serving as the body’s first line of defense which initiates both innate and adaptive immune responses in many clinical conditions of health and disease. Our innovative course design was developed utilizing this natural connection between immunology and HEENT to integrate them by concurrently applying principles of immunology in the context of HEENT case scenarios. These cases represented clinical conditions commonly seen in primary healthcare, as HEENT conditions, particularly upper respiratory tract infections, ranked as one of the top reasons for primary care physician office visits in the USA and the world with a score of 15.2/20 (score 20 representing the top ranked reason for visit) [7]. Active learning techniques including case-based learning (CBL) and small group discussion (SGD) were purposefully employed for the integrative model to increase student engagement as well as to enhance the contextualization and application of the content [8–11].

Using this pioneering course as a model, we hypothesized integrating immunology and HEENT through active learning activities would be an achievable approach to building an integrative course supporting the learning of both basic and clinical sciences. In this study, we utilized multiple methods to evaluate student perceptions of this novel curriculum design.

## Materials and Methods

### Course Model Design

A six credit-hour Immune System and HEENT course was offered to the inaugural class during their first year in the spring semester 2021 at SHSU-COM. The course was developed by a team comprised of an immunologist, a microbiologist, a primary care physician, a pathologist, and a pharmacologist, which allows for a cross-disciplinary collaboration. To tie together concepts and applications related to the two subjects, we deliberately reviewed associated topics from COMLEX-USA (Comprehensive Osteopathic Medical Licensing Examination of the United States) master blueprint [12, 13] and the USMLE (United States Medical Licensing Examination), [13] content outline. We categorized immune system topics by their manifestations in HEENT: mechanical, inflammatory, infectious, and neoplastic. These topics present great opportunities for us to review the most common problems presenting to the rural primary care physician’s office and integrate the clinical presentation with associated HEENT structure and underlying immune function. A blended teaching methodology was employed to deliver the course. Each week, concepts related to HEENT and immunology was delivered separately either online or in class. These concepts were reconsolidated in a weekly CBL/SGD session conducted on Zoom^®^. Each 2-h face-to-face CBL/SGD session was facilitated by a scientific and clinical facilitator pair. Seventy-four enrolled students worked in pre-defined groups of 6–8 per session. Groups remained constant throughout the course to facilitate the development of team dynamic. In these sessions, students in small groups were provided a clinical case and a list of clinically driven questions and learned to apply foundational immunology in primary care–focused clinical context, while practicing critical thinking through analyzing clinical presentations and laboratory results for differential diagnosis. Faculty facilitators from both immunology and primary care specialties were present as facilitators to provide instant feedback. Although the weekly CBL/SGD session was focused on correlation of HEENT with the immune system, other disciplines such as pathology and pharmacology were integrated through this learning opportunity as well. At the end of each session, students were required to submit a Google^®^ Form to report their discussions on those questions. The weekly planning for topics integration is provided in Appendix 1 Table 2. An example of weekly case is provided in Appendix 2.

In addition to the group report, course assessments also included readiness quizzes for both immunology and HEENT sessions, TBL quizzes, and a final exam. Pre-tests were originally planned; however, due to extenuated weather condition which caused cancellation of the first week of course at the time, they were not implemented.

### Mixed Methods for Evaluating Student Perception

As a brand-new course for the inaugural class, no comparison study was performed, and the evaluation was focused on student perception of the integrative model instead of learning outcomes since all students successfully passed the course. Proponents of mixed methods believe that using both quantitative and qualitative methodology in a single research study potentially strengthens the study [14]. In addition, using more than one approach in qualitative data analysis, as recommended by Leech and Onwuegbuzie [15], can increase interpretive validity, or the degree to which the perspectives of participants are accurately rendered by the researcher [16]. Likewise, using more than one qualitative approach can allow researchers to gain a deeper understanding of participants’ perspectives [17].

#### Quantitative Data Analysis

We administered to the inaugural class of 74 first-year students a voluntary 12-item survey that included five demographic items and five rating-scale items. Demographic items addressed participants’ age range, gender identification, grade-point average (GPA), and previous learning experience with immunology. Four rating-scale items asked participants to reflect on the degree to which the integration of HEENT and immunology in the weekly CBL/SGD helped them solidify their learning, provided them with experiences to develop critical thinking skills, improved their confidence in applying their learning to answering clinical questions, and improved their confidence in taking a comprehensive approach to differentially diagnose HEENT diseases. An additional rating-scale item asked participants to rate the overall effectiveness of CBL/SGD sessions in integrating HEENT and immunology. Descriptive statistics were calculated for each of the five rating-scale items.

#### Qualitative Data Analysis

In addition to administering the survey, we conducted a focus group of 10 students selected via convenience sampling. Qualitative data consisted of answers to the two open-ended survey items and the focus group transcript. To prepare for analysis, we first de-identified the focus group transcript and corrected any transcription errors. Next, we emailed focus group members, inviting them to schedule a brief Zoom^®^ meeting to engage in member checking by reviewing their individual contributions to the focus group [18]. No focus group participants chose to take advantage of this opportunity, even though we extended the option to meet outside of normal business hours if needed. Last, we analyzed the focus group transcript data and the data from the open-ended survey questions using two different approaches: constant comparison analysis [19] and classical content analysis [20].

To begin constant comparison analysis, we reread the de-identified, corrected focus group interview transcript and used Dedoose 8.3.47b to assign 38 open in vivo codes (preliminary codes containing participants’ own words) to the text [21]. We repeated this step with the responses to the open-ended survey questions, assigning 42 open in vivo codes to the text. After we reviewed the accuracy and relevance of these codes and used the software to merge similar codes and remove other codes that no longer seemed pertinent, 32 codes remained assigned to the focus group transcript and 29 codes remained assigned to the open-ended survey responses. The 32 codes assigned to the focus group transcript were sorted into nine different categories, which were then combined into three broad themes. The 29 codes assigned to the open-ended survey responses were sorted into eight different categories, which all fit within the previously identified themes: student satisfaction with integration of HEENT and immunology, content and time involved in CBL/SGD, and suggestions for improvement. Last, we used printouts from the software to conduct classical content analysis, calculating percentages of codes associated with each theme to determine their relative significance to the participants.

## Results

### Quantitative Results: Participants Considered Effectiveness of Integration Average

Although 54 of 74 students agreed to participate in the survey, only 48 of these participants answered demographic items and 39 of these participants answered rating-scale items (53% response rate). As mentioned previously, demographic items addressed participants’ age range, gender identification, GPA, and previous learning experience with immunology. Participants were approximately evenly divided between those who identified as male (*n* = 25) and those who identified as female (*n* = 23), which is similar to the class with 40 males and 34 females. The majority of participants ranged in age from 18 to 25 years (*n* = 33) and possessed undergraduate overall GPAs ranging from 3.6 to 3.8 (*n* = 26) and undergraduate science GPAs ranging from 3.0 to 3.5 (*n* = 22). The vast majority of participants had either no experience (*n* = 23) or only a little experience (*n* = 23) with immunology prior to taking this course.

Regarding rating-scale items, nearly half (48.72%) of participants rated the overall effectiveness of using CBL/SGD for system integration (1–very ineffective, 5–very effective) as average, with a mean value of 3.08 (std 0.86). Mean responses concerning the degree to which the integration of immunology and HEENT in the weekly CBL/SGD helped solidify learning, provided experiences to develop critical thinking skills, improved confidence in applying learning to answering clinical questions, and improved confidence in taking a comprehensive approach to differentially diagnose HEENT diseases (1–not at all, 6–perfectly) ranged from fairly well (3) to quite well (4). Mean responses to these rating-scale items are depicted in Table 1.

**Table 1:** Students’ perceptions of the integration of immune system and HEENT

**Rating-scale items**	**Means**	**Standard deviations**
How well does the integration of HEENT and immune system in the weekly case-based learning in small group sessions…		
Help you solidify the learning of immunology within the context of HEENT clinical scenarios?	3.05	1.18
Provide experiences to develop your skills in critical thinking?	3.62	1.19
Improve your confidence in integrating and applying HEENT and immunology to solve clinical questions?	3.33	1.21
Increase your confidence in taking a comprehensive approach to differentially diagnose HEENT diseases and understand their pathogenicity?	3.41	1.30

### Qualitative Results: Participants Held Mixed Perceptions and Voiced Suggestions for Improvement

#### Student Satisfaction with Integration of HEENT and Immunology

Associated with almost half (47.5%) of all assigned codes, the theme *student satisfaction with integration of HEENT and immunology* seemed most significant to research participants who provided qualitative data. Codes that related to positive perceptions of satisfaction (e.g., “integration made sense”) comprised 23% of all assigned codes, and codes that related to negative perceptions of satisfaction (e.g., “a force to integrate HEENT and immunology”) comprised 25% of all assigned codes. However, survey participants appeared to focus their comments primarily on positive perceptions, while focus group participants appeared to comment more negatively.

This theme included codes related to students’ positive perceptions of the integration of the two topics. Survey participants wrote comments such as “the coursework and lectures were great,” “enjoyed the integration,” and “guided questions helped.” One survey participant elaborated further, stating “I felt it worked well to integrate HEENT and immuno[logy]. They overlapped and branched out to let us see how medicine is all interrelated.” In response to what was most beneficial pertaining to the integration of immunology and HEENT, a focus group participant stated the following:

“I guess in terms of board studying, you need to study everything at once, and it’s not so, like, broken down. Like neuro, HEENT, immunology, so I guess we get a taste of that in like, everything was thrown at you at once, and you just have to answer the questions that you’re getting.” (Student 5)

The other codes included in this theme related to students’ negative perceptions of the integration of HEENT and immunology, including a lack of repetition of concepts. One focus group participant mentioned that this effort was a “great attempt at” integration, but ultimately “it just wasn’t enough.” (Student 9) Although a couple of focus group participants referenced the importance of integration, they, along with other focus group participants, also held negative perceptions regarding this specific attempt at integration. A focus group participant remarked that “the integration of HEENT and immunology was quite subpar, and it was very limited.” (Student 8) A second focus group participant said that “I don’t feel like the integration of immunology and HEENT was reviewed very much during the course.” (Student 7) A third participant alluded to lack of review/repetition of concepts by suggesting breaking “up the HEENT lectures so it wasn’t just five really big HEENT lectures, and then we just never see it again,” (Student 3) as well as with the following comment:

“I think if it had been scheduled a lot better in a way that we constantly also got HEENT as well as immuno[logy]…... Then that would have been better, instead of them just kind of last minute throwing a lot of information at us.... ... At that point the integration even having both subjects together kind of became useless to me?” (Student 3)

Codes in this theme related to students’ negative perceptions of the integration of HEENT and immunology also described a “force to integrate” the material. Two survey participants remarked that they felt as if they were taking two different classes, and three focus group participants referenced a feeling of “bouncing” between topics (e.g., “felt like I was bouncing between science and clinical” [Student 5]). Related, a focus group participant had trouble determining how to study these topics:

“I think another challenge was also how we mentioned that there were only five HEENT lectures, and then the percentage of our final exam that HEENT was involved in wasn’t that much. But at the same time, it was so much information that I was kind of at this like, do I study immunology more or HEENT more, and it was like I didn’t know where to like, spend my time.” (Student 10)

#### Content and Time Involved in CBL/SGD

Associated with 32.8% of all assigned codes, the theme *content and time involved in CBL/SGD* was next most significant to research participants who provided qualitative data. Focus group participants and survey participants seemed approximately equally concerned with the degree of helpfulness of CBL/SGD and the degree of complexity of CBL/SGD. However, survey participants appeared more concerned than focus group participants with the length of time devoted to CBL/SGD, and focus group participants appeared more concerned than survey participants with expanding the content of CBL/SGD in some way.

This theme included codes that described the degree of helpfulness of CBL/SGD. For example, five different survey responses referenced CBL/SGD as “helpful,” beneficial,” or “effective.” A survey participant also noted that CBL/SGD questions regarding specific immunology pathways were a “great review” of immunology material. A focus group participant appreciated how CBL/SGD helped them “think through some of these processes,” (Student 4) and another mentioned that CBL/SGD provided them “help with a differential diagnosis.” (Student 8) The latter participant and another focus group participant also considered the use of Google Docs helpful. Likewise, survey participants mentioned the benefits of the use of Google Docs (e.g., for studying and reviewing) in four different responses. However, a survey participant who deemed the use of Google Docs beneficial also noted that “the quality of work was dependent on the group,” with some students in some weeks participating very little or not at all. A couple of other survey participants went as far as to declare CBL/SGD “unnecessary” and “a waste of time.” One focus group participant noted the unnecessary repetitiveness for integrating immunology in the context of HEENT:

“When you were doing integration for like immunology, would be like what kind of steps does your body take to do these processes? And a lot of the times, the process was like the same. Like we were either talking about the innate or adaptive immune system.” (Student 3)

In addition, one focus group participant commented that it “didn’t really seem to make sense” (Student 7) to have four groups discuss the same disease, and survey participants made similar comments in three different responses, such as “more discussion over several cases would have allowed students to solidify the learning of immunology within HEENT context clinical scenarios.”

This theme also included codes that referred negatively to the length of time devoted to CBL/SGD, with similar negative remarks in 10 different survey responses and from three different focus group participants. Open-ended survey responses included phrases such as “a bit long,” “drawn out in some cases,” and “time-consuming” to refer to CBL/SGD. Focus group participants seemed to doubt the usefulness of the length of time allotted for CBL/SGD given the content. For example, one focus group participant commented “I don’t think it was useful spending two hours on, like, a simple case of otitis media, for instance. It doesn’t make sense.” (Student 5) Another focus group participant agreed “that it did not need to be 2 h,” elaborating that “a lot of it could have been done with 1 h.” (Student 1) A third focus group participant concurred that “having like a simple case like that in two hours wasn’t really like the best use of our time.” (Student 10).

Other codes included in this theme alluded to the degree of complexity of CBL/SGD content. Although a couple of focus group participants and a couple of survey responses referenced preparing for CBL/SGD, four focus group participants indicated engaging in little to no preparation. One focus group participant connected their lack of preparation in part to the lack of complexity of the CBL/SGD content:

“I wouldn’t prepare just because I didn’t have time. And I was able to do the case without any background knowledge, so it just kind of showed that it was a very simple case, but like, I didn’t necessarily need to prepare for?” (Student 10)

Survey participants indicated satisfaction with the complexity of CBL/SGD content in a couple of responses (e.g., “I was challenged and it did require me to use critical thinking”), but five survey responses referred to the lack of complexity of CBL/SGD content. For example, one respondent wrote “a lot of the groups were just looking at the presented lectures and taking information. There was not much critical thinking that the students completed throughout the entire 2 h.” Other survey participants noted that “SGD cases were not very detailed” or “too simple.” One survey participant wrote that, “in most sessions, students had answered the questions fairly quickly,” and another believed “it would’ve been more effective if either the sessions were shorter or the cases were more complex.”

Related to this desire for complexity was a desire for expanding CBL/SGD content in some way, and codes related to expansion comprised the remainder of codes for this theme. Some focus group participants wanted more faculty involvement in the process, as illustrated by one participant who commented that “one tradition that could possibly be taken from [another] block was how the professors also did case presentations” (Student 1) following lecture.

Another focus group participant suggested that faculty provide “a professor-reviewed document on each of the cases…[with] expanded information,” (Student 8) and one of the survey responses referenced a similar idea. Other suggestions from focus group participants for expanding CBL/SGD content included “expanding on like the pharmacology and also on the differential diagnosis,” (Student 8) “a little bit more info on…pathophysiology of…the disease,” (Student 8) and “more in detail into treatments or…all the different disorders and diseases we’d see in that region of the body.” (Student 10).

#### Suggestions for Improvement

Associated with 19.7% of all assigned codes, the theme *suggestions for improvement* seemed least significant to research participants who provided qualitative data. Regarding this theme, comments from focus group participants generated twice as many codes as comments from survey group participants. Therefore, focus group participants appeared more concerned with providing suggestions for improvement than survey participants.

This theme included codes that described suggestions for improving the integration of HEENT and immunology. One focus group participant recommended having “more practice questions and having [faculty] talk through them.” (Student 6) This participant also referenced spreading HEENT material into more and shorter lectures, rather than “3-h super long” lectures “with a whole bunch of stuff piled in.” (Student 6) Another participant elaborated further, musing that integration might have been better achieved if “big chunks of [HEENT] information…had been more spread out.” (Student 3).

Paradoxically, three focus group participants seemed to want to pull HEENT and immunology even further apart as a way to improve integration. For example, one focus group participant indicated that further separation would help with the “bouncing” issue:

“I think it would have just been maybe easier to have HEENT placed somewhere else or have its own week, or like, a dedicated time period where you can focus on this, like, material just because of how important it is. And then, I guess…I don’t want to say scale back on the immunology because maybe it is all important but, I guess integrate it in a way where it’s more blocked off, so that you’re not doing the bouncing in and out.”(Student 4)

Another participant suggested “you can do… two or three weeks of…immunology, and then the last ones are like HEENT,” (Student 3) and a survey response was similar, stating “it may have been easier to digest the material if it was split with all the immunology at once and then all the HEENT.”

The other codes included in this theme described suggestions for different integration pairings. One focus group participant thought that HEENT would have paired better with clinical medicine. One survey response referenced that both HEENT and immunology should have been included in microbiology, and seven focus group participants recommended having “HEENT placed somewhere else” (Student 4) or pairing either HEENT or immunology with microbiology instead of pairing the two together. One advocate for pairing HEENT with microbiology made the following comment:

“Maybe if it was more integrated during our microbiology course? Because I feel like a lot of that stuff related directly to the viruses and the clinical presentations, that we were learning. But when we were studying microbiology, it was purely just the mechanisms behind, like, the virus, for example. So, if they are more integrated in that this [is] the virus, this is how it works, and here are the clinical presentations of the eyes, mouth, nose, like that would have made a little bit more sense for us.” (Student 6)

One of these focus group participants also speculated that HEENT should not be integrated with any one area:

“Tying HEENT to one specific block doesn’t really do it the service that it needs? Because it’s going to show up again and again, like, when we do cardiopulmonary, HEENT is going to become a big factor again. Like when we do GI specifically, nose, throat, mouth, like all that comes back in. So it’s like, limiting it to just immunology and then never seeing it again in terms of just the anatomy the physiology kind of takes away from our learning, I guess? Because then we’re just learning diseases, and…it’s less on the pathophysiology as to how these diseases come about.” (Student 7)

Another advocate for an alternative pairing wanted “to make a case on integrating microbio[logy] and immunology,” (Student 5) and a survey response indicated a similar idea: “I believe immunology might be integrated more seamlessly with course like microbiology, since it seems like those two courses together is a more accurate representation of what our boards questions will be like.”

## Discussion

Undergraduate medical education in the USA and world has moved from Flexner’s discipline-based curriculum to integrative curriculum which helps students to see the relevance of basic and biomedical sciences applied to clinical practice at an earlier stage of training [22]. Although most medical schools have moved to a system-based integrative curriculum, the foundational sciences, such as immunology, are still often offered outside the system course series as a focused content area at the beginning of medical school. This approach allows students to fully grasp the basic principles of immunology before introducing the more complex aspects of medicine in systems. However, teaching immunology alone may also limit students’ ability to apply learned concepts in clinical examples, particularly when multiple systems are involved in disease state, due to minimal or no clinical exposure. Combining different disciplines and systems as referred by integrated curriculum provides a reiterative process that can help students consolidate information they are required to know, apply knowledge through a more holistic approach, and enhance the retention of learned concepts [2]. In addition, thoughtful integration of basic science with clinical observations and laboratory findings is needed to fill the gap that exists between the knowledge obtained in preclinical years and the more practical, skill-based reasoning that medical students acquire during clinical rotations [23]. In contrast to traditional stand-alone foundational science courses and systems courses in which body systems are taught independently, our new course design presented a novel approach for integration of foundational and clinical sciences and of multiple body systems by concurrently presenting them in the context of clinical cases.

Results of this mixed methods study showed that students perceived some advantages of the integration employed. They reported that the integration helped their critical thinking skills and improved their ability to develop differential diagnosis. Our method of integration was grounded in adult learning theory, or andragogy. First, students are willing to invest time into learning a particular topic only when they understand its actual relevance to their own lives [24]. Second, students learn and apply when they can organize knowledge to make rich connections between important and meaningful concepts and principles [25]. Combining HEENT and immunology introduced overlapping clinical scenarios where a multitude of different bodily systems were affected. It allowed for the instructors to have a constant clinical tie-in that could help students anchor their learned knowledge to a larger aim [25]. Our module also employed active learning methods including CBL and SGD to provide student-driven learning. CBL has been widely used in healthcare education and approved to be an effective pedagogical method [9]. SGD helps students develop advanced team-work skills which are essential in a modern healthcare environment [26, 27]. Presenting clinical examples in SGD and CBL requires students to make personal connections to a given topic that further enhances retention and understanding by making the learning an active process. Learning becomes more intimate and necessary rather than simply learning for learning’s sake. In a medical school setting, this undoubtedly assists in helping students build a stronger repertoire for developing differential diagnoses as well as collaborative skill.

In our study, students presented several perspectives for the improvement of the integrative model. Length and complexity of CBL/SGD sessions were the major concerns in our study. Although a systemic review found the shortest intervention for CBL was 2 h [9], which was the time we used for our session, the students felt 2 h for each CBL/SGD session was too long. This might be due to perceived lack of complexity of the case scenario and the related activities. Our finding aligned with other studies where the students preferred more structure and clearer instructions and learning tasks for CBL [28, 29]. More discussion over several cases and expanding integration of other disciplines might also be helpful per students’ feedback. Faculty facilitators/tutors’ engagement was another thing brought up by the students. Although part of it might be caused by the sudden transition to online learning due to COVID, there are many methods to improve the facilitation of the small group discussion in the future both in person and online [26]. Together, these strategies may help students solidify the learning of integrative knowledge.

In addition to this, students also felt that there was an imbalance of content from the two subjects, with a much larger amount of immunology over HEENT. This imbalance created a sense of tension for students that felt like the two topics were forced into one merged course, making a primary focus difficult to discern. This contributed to the feeling of uneasy integration and confusion regarding the course’s primary focus, especially during exam preparation. Immunology and HEENT were not successfully and fully integrated in this course partially because the rollout and instruction of the two courses required vastly different approaches. This made it difficult to teach the two topics interchangeably. Immunology requires more explanation of the pathophysiologic pathways and activation factors foundational to immunology. HEENT complications can range from oncologic issues to infectious disease topics, depending more heavily on case presentations and descriptive care plans with treatment management. Separating the two topics into individual sessions, with HEENT as a singular lecture series each week, may have contributed to the student’s sense of discord within this novel course. Further integration and cohesion should be pursued in future iterations of combined courses such as this one. Reaching a point of true interdisciplinary learning requires much communication and joint planning so that the objectives for each topic introduced in the course are given an adequate amount of space and time for learners. Additionally, finding subjects in medicine that better relate, such as microbiology and immunology, might be the better solution since these courses tend to have more interrelated topics that are naturally cohesive [30]. The goal of combining different fields of medicine should be focused on creating a synergistic course that results in proficiency in both topics while not sacrificing learning for the sake of trimming off some time in the curriculum. If this method is done successfully, lessons and assessments would not be identified as one topic or the other; successful integration would make the course feel unified with multiple subjects present in each lesson plan.

As a pioneer course offered to the inaugural class, the in-house comparison of this course design with traditional course design in student learning outcomes was not feasible. Since students were in their first year of medical education, it was too early to assess the long-term impact of learning from this course on their clinical performance. Bearing these limitations in mind, we used a mixed method approach, which employs both quantitative and qualitative analyses to evaluate the students’ perceptions of the course design for future course improvement. This method revealed a more robust and comprehensive picture of student attitudes towards course integration and learning outcomes. Although Likert scales are a preferred evaluation instrument for sociological quantitative research, they also produce an element of bias towards responses, skewing data [31]. However, the inclusion of qualitative methods provided additional information that allowed us to gain greater perspective. For example, if we had collected and analyzed only quantitative survey data, our study may have concluded that participants held fairly positive perceptions regarding the integration of HEENT and immunology, since nearly half of participants who answered rating-scale items rated the overall effectiveness of using CBL/SGD for system integration to be average. In addition, mean responses to remaining rating-scale items reflected agreement that the integration of HEENT and immunology promoted desirable outcomes to some degree (e.g., helped solidify learning). However, analysis of qualitative data from both the open-ended survey questions and the focus group transcript provided more nuanced information, which modified our perspective. As described in “Materials and Methods,” two qualitative analysis types were employed for this study: constant comparison analysis and classical content analysis. Via classical content analysis, we learned what seemed most important to participants collectively; via constant comparison analysis, we learned how participants’ perceptions varied.

This in-depth analysis of the survey and focus group qualitative data helped us develop valuable recommendations for health professions curriculum developers interested in creating an integrative systems-based course or adding integrative learning activities within medical curricula. These recommendations include (1) developing more complex case modules for SGD sessions to enhance content integration, (2) being more cognizant of group activities’ time allocation and usage, and (3) being more intentional about which systems curricula to group together for maximal student learning outcomes, such as integrating immunology and microbiology curricula. Additionally, using mixed methods is an invaluable tool to assess the curricular efficacy and student outcomes. Multiple curriculum development models emphasize the importance of evaluation in the iterative process of curriculum development, as well as an appropriately calibrated evaluation instrument in the feedback process [32, 33]. End-of-course evaluations are a common tool to evaluate the overall course effectiveness; however, they are often times too broad to identify the efficacious aspects in course design. Using questionnaires and focus groups targeting the evaluation of specific course components is beneficial to gain thorough feedback that could promote improved curricular design in the future. If evaluation resources are limited (i.e., lack of a qualitative study expert or time for adopting multiple evaluation methods), adding targeted questions for specific course design elements within an end of course survey may address this challenge.

Our study has several limitations. As a pioneer course for the inaugural class, there was more liberty for innovative curricular design; however, we lacked a control group to compare and demonstrate the direct impact of this course design on student learning outcome. The course was offered in the second semester of the preclinical curriculum as the second system course. Although the CBL/SGD was to provide opportunities to apply integrative information, our students lacked both foundational and clinical knowledge and skill for application in their first year of medical school. It was also too early to assess the application of the integrative knowledge in students’ clinical performance.

Despite of these limitations, our model suggests that it is feasible to develop and implement novel integrative curriculum designs, with careful thought and consideration. Our future direction includes reconstructing the curriculum so that immunology is paired with a more appropriate course, such as microbiology, and that HEENT is integrated throughout the students’ clinical skills course and their other organ system courses.

## Conclusions

Our course model provides insights into the development process of an integrative course in healthcare professional programs. Through this experience, we gained a deeper understanding of how students perceived the integration of the two targeted subjects using active learning format and learned the importance of using multiple and mixed methods to evaluate the curriculum design which can support course improvement.

## Data Availability

The datasets used and/or analyzed during the current study are available from the corresponding author on reasonable request.

## Appendix 1

**Table 2:** Weekly curricular planning for topics integration of immunology and HEENT

**Week**	**Immunology topic**	**HEENT topic**	**Integrated cases of SGD/CBL and their main learning objectives**
1	Innate immune system	Eye	Conjunctivitis 1. Determine a differential diagnosis for red eyes 2. Relate concepts pertaining to innate immune barrier and immune privilege to diseases associated with eyes
2	Adaptive immune system	Throat	Streptococcal pharyngitis 1. Determine a differential diagnosis for sore throat 2. Relate concepts pertaining to adaptive immunity and immune memory to diseases associated with throat 3. Apply knowledge of immunodiagnostics to explain the principle of rapid Strep testing
3	Mucosal immunity and immunodeficiency	Ear	Otitis externa 1. Determine a differential diagnosis of ear pain 2. Relate concepts pertaining to the role of normal microflora in immunity to diseases associated with ear
4	Autoimmunity and hypersensitivity	Nose	Allergic rhinitis 1. Determine a differential diagnosis for nose congestion 2. Relate concepts pertaining to hypersensitivity reactions to diseases associated with nose 3. Apply immunological concepts to explain the principles of allergy treatment
5	Tumor and transplantation immunology	Head and neck	Tobacco-associated oral cancer 1. Determine a differential diagnosis for head and neck mass 2. Relate concepts pertaining to tumor immunology to diseases associated with head and neck 3. Explore immunotherapy as treatment options for head and neck cancers

## Appendix 2 Example of SGD/CBL Session

### Students will First Learn the Following Topics in Separate Sessions Before SGD/CBL Session:

Immune system: Innate immunity, overview of adaptive immune system, T cell-medicated immunity, humoral immunity

HEENT: Throat

### Students will then Participate in this SGD/CBL: Disorders of Throat

#### Case: A 7-Year-old Boy with CC: “Sore Throat”

A fully vaccinated patient is brought by mother, who reports 2 d of “fever” (up to 101.5F) and painful swallowing. He is able to swallow liquids and has been consistently drinking water and eating yogurt and ice cream

Review of systems is significantly negative for runny nose, cough, nausea, vomiting, diarrhea, chills, neck stiffness, and travel. Review of systems is significantly positive for exposure to a 5-year-old neighbor last week with the same symptoms

Exam:

Temp 38.3 °C (101 °F), HR 90/min, RR 18/min, BP 118/68 mmHg

The child is sitting upright on the table reading a story book. He is not ill-appearing

Examination of eyes, ears, and nose is unremarkable

Examination of the throat:

Neck exam reveals bilateral submandibular and anterior cervical tender and enlarged lymph nodes that are < 1 cm in size

Abdominal exam reveals no hepatosplenomegaly

Skin reveals no rash

## Small Group Discussion:

1. Create a problem list for this patient.
2. Develop a brief primary care differential diagnosis for this patient’s problem list.
3. What is the most likely cause of your patient’s sore throat? Why? What other physical findings might you see in a patient with this diagnosis?
4. Describe your patient’s immune response to the etiologic agent(s) and explain how the immune response is associated with this patient’s clinical presentation.
5. Provide an explanation for why your patient’s immune system defenses were not successful in preventing this.
6. Propose a management plan for your patient. Include any pertinent osteopathic manipulative medicine or home-care instructions. Would you include antibiotics in your plan? Why or why not?
7. What other diagnosis and management plan would you have considered if your patient had also had runny nose and cough? Inability to swallow? Relate immune mechanisms to each alternative diagnosis.
8. How would the following affect your management plan:
  - Presence of cough and coryza?
  - Ill-appearance and drooling?
  - The presence of vesicles in the mouth or throat (and perhaps on the hands and feet)?
  - Lack of vaccination and travel to S. America?
9. Any anticipatory guidance that you want to give the mother?

## References

[1] How medical education is changing, policy priorities to improve our nation’s health. https://www.aamc.org/system/files/c/2/472906-howmedicaleducationischanging.pdf. Accessed 10 May 2020.

[2] Brauer DG, Ferguson KJ. The integrated curriculum in medical education: AMEE Guide No. 96. Med Teach. 2015;37(4):312–322. 10.3109/0142159X.2014.970998. 10.3109/0142159X.2014.970998 25319403

[3] LCME Annual Medical School Questionnaire Part II, 2017–2018. https://www.aamc.org/data-reports/curriculum-reports/report/curriculum-reports. Accessed 10 Feb 2020.

[4] Bansal AS (1997). Medical students’ views on the teaching of immunology. Acad Med.

[5] Lee A, Malau-Aduli B. Medical students’ learning experiences and perceptions of immunology. Int J Med Educ. 3(1):1–12.

[6] DeZee KJ, Artino AR, Elnicki DM, Hemmer PA, Durning SJ (2012). Medical education in the United States of America. Med Teach.

[7] Finley CR, Chan DS, Garrison S (2018). What are the most common conditions in primary care?. Systematic review Can Fam Physician.

[8] Brown PC (2014). Make it stick : the science of successful learning.

[9] Thistlethwaite JE, Davies D, Ekeocha S, et al. The effectiveness of case-based learning in health professional education. A BEME systematic review: BEME Guide No. 23. Med Teach. 2012;34(6):421–444. 10.3109/0142159X.2012.680939. 10.3109/0142159X.2012.680939 22578051

[10] Ferreri SP, O’Connor SK (2013). Redesign of a large lecture course into a small-group learning course. Am J Pharm Educ.

[11] Annamalai N, Manivel R, Palanisamy R (2015). Small group discussion: students perspectives. Int J App Basic Med Res.

[12] NBOME. COMLEX-USA master blueprint. https://www.nbome.org/Content/Exams/COMLEX-USA/COMLEX-USA_Master_Blueprint_2018-2019.pdf.

[13] Step 1 Content Outline and Specifications | USMLE. https://www.usmle.org/prepare-your-exam/step-1-materials/step-1-content-outline-and-specifications. Accessed 7 July 2022.

[14] Johnson B, Christensen L (2012). Educational research: quantitative, qualitative, and mixed approaches.

[15] Leech NL, Onwuegbuzie AJ (2007). An array of qualitative data analysis tools: a call for data analysis triangulation. Sch Psychol Q.

[16] Maxwell JA. Understanding and validity in qualitative research. In: The qualitative researcher’s companion. SAGE Publications, Inc. 2022. 10.4135/9781412986274.

[17] Sechelski A, Onwuegbuzie A. A call for enhancing saturation at the qualitative data analysis stage via the use of multiple qualitative data analysis approaches. TQR. 10.46743/2160-3715/2019.3554. Accessed 17 April 2019.

[18] Manning K (1997). Authenticity in constructivist inquiry: methodological considerations without prescription. Qual Inq.

[19] Glaser B (1965). The constant comparative method of qualitative analysis. Soc Probl.

[20] Berelson B (1952). Content analysis in communication research. Ann Am Acad Pol Soc Sci.

[21] Saldaña J (2013). The coding manual for qualitative researchers.

[22] Irby DM, Cooke M, O’Brien BC (2010). Calls for reform of medical education by the Carnegie Foundation for the Advancement of Teaching: 1910 and 2010. Acad Med.

[23] Educating doctors to provide high quality medical care: a vision for medical education in the United States. Report of the Ad Hoc Committee of Deans. AAMC.; 2004. https://www.yumpu.com/en/document/read/24704909/educating-doctors-to-provide-high-quality-medical-care-aamcs-. Accessed 8 Dec 2022.

[24] Knowles M. The modern practice of adult education: from pedagogy to andragogy. Revised Edition. Cambridge Book Co; 1988.

[25] Ambrose SA, Bridges MW, DiPietro M, Lovett MC, Norman MK, Mayer RE. How Learning works: seven research-based principles for smart teaching. Wiley; 2010.

[26] Edmunds S, Brown G. Effective small group learning: AMEE Guide No. 48. Med Teach. 2010;32(9):715–726. 10.3109/0142159X.2010.505454. 10.3109/0142159X.2010.505454 20795801

[27] van Diggele C, Burgess A, Mellis C (2020). Planning, preparing and structuring a small group teaching session. BMC Med Educ.

[28] Hilgenberg C, Schlickau J (2002). Building transcultural knowledge through intercollegiate collaboration. J Transcult Nurs.

[29] Hakkarainen P, Saarelainen T, Ruokamo H. Towards meaningful learning through digital video supported, case based teaching. Australas J Educ Technol. 2007;23(1). 10.14742/ajet.1275.

[30] Smith JM, McClelland EE (2012). Teaching immunology through microbiology. Med Sci Educ.

[31] Pimentel J (2019). Some biases in Likert scaling usage and its correction. Int J Scis: Basic Appl Res.

[32] Bhuiyan P, Supe A, Rege N. The art of teaching medical students - e-book. Elsevier Health Sci. 2015.

[33] Lunenburg FC. Curriculum development: inductive models. Sch. 2011;2(1).

